# Targeted sequencing reveals candidate causal variants for dairy bull subfertility

**DOI:** 10.1111/age.13089

**Published:** 2021-05-24

**Authors:** R. Abdollahi‐Arpanahi, H. A. Pacheco, F. Peñagaricano

**Affiliations:** ^1^ Department of Animal Sciences University of Florida Gainesville FL USA; ^2^ Department of Animal and Dairy Sciences University of Wisconsin‐Madison Madison WI USA

**Keywords:** causal mutation, DNA sequencing, sire conception rate

## Abstract

Bull fertility is a key factor for successful reproductive performance in dairy cattle. Since the semen from a single bull can be used to inseminate hundreds of cows, one subfertile bull could have a major impact on herd reproductive efficiency. We have previously identified five genomic regions, located on BTA8 (72.2 Mb), BTA9 (43.7 Mb), BTA13 (60.2 Mb), BTA17 (63.3 Mb), and BTA27 (34.7 Mb), that show large dominance effects on bull fertility. Each of these regions explained about 5–8% of the observed differences in sire conception rate between Holstein bulls. Here, we aimed to identify candidate causal variants responsible for this variation using targeted sequencing (10 Mb per region). For each genomic region, two DNA pools were constructed from n≈20 high‐fertility and n≈20 low‐fertility Holstein bulls. The DNA‐sequencing analysis included reads quality control (using FastQC), genome alignment (using BWA and ARS‐UCD1.2), variant calling (using GATK) and variant annotation (using Ensembl). The sequencing depth per pool varied from 39× to 51×. We identified a set of nonsense mutations, missense mutations, and frameshift variants carried by low‐fertility bulls. Notably, some of these variants were classified as strong candidate causal variants, i.e., mutations with deleterious effects located on genes exclusively/highly expressed in testis. Genes affected by these candidate causal variants include *AK9*, *TTLL9*, *TCHP*, and *FOXN4*. These results could aid in the development of novel genomic tools that allow early detection and culling of subfertile bull calves.

Service sire has been recognized as an important factor affecting herd fertility in dairy cattle, influencing not only the fertilization process, but also the viability of the embryo during early stages of development (Ortega *et al*. 2018). Dairy bull fertility can be evaluated in the laboratory using different semen production and quality attributes, or in the field using conception rate records. The US dairy industry has had access to a national phenotypic evaluation of male fertility called sire conception rate (Kuhn & Hutchison 2008). This bull fertility evaluation is based on a large, nationwide database of confirmed pregnancy records. Interestingly, there is a remarkable variation in sire conception rate among dairy bulls, with more than 10% conception rate difference between high‐fertility and low‐fertility bulls (Han & Peñagaricano 2016; Rezende *et al*. 2018). Our group has been investigating potential genetic factors underlying the observed variation in sire conception rate. In fact, we have identified five genomic regions, located on BTA8 (72.2 Mb), BTA9 (43.7 Mb), BTA13 (60.2 Mb), BTA17 (63.3 Mb), and BTA27 (34.7 Mb), showing dominance effects on sire conception rate (Fig. 1; Nicolini *et al*. 2018; Nani *et al*. 2019). Notably, each of these five regions explained about 5–8% of the observed differences in sire conception rate between Holstein bulls (Fig. 1). Although these regions harbor several genes with known roles in sperm biology, the causal variants (causal mutations or QTNs) responsible for these effects remain unknown. As such, the objective of this study was to identify candidate causal variants responsible for these large non‐additive effects. This research will aid in the development of tools to assist dairy breeders make accurate management and selection decisions on male fertility, such as the early culling of predicted subfertile bull calves.

**Figure 1:**
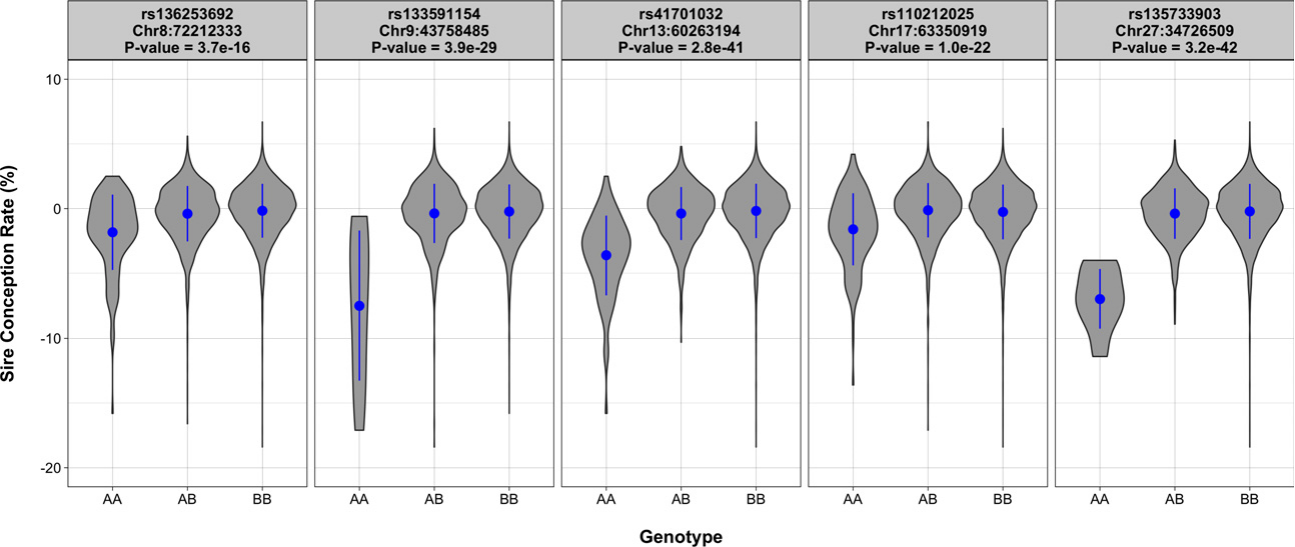
Genomic regions with large dominance effects on sire conception rate in US Holstein bulls. Violin plots showing the observed sire conception rate records for each genotype of the five significant markers (Adapted from Nani *et al*. 2019).

For each genomic region, two pools of DNA were constructed from roughly n≈20 high‐fertility and n≈20 low‐fertility Holstein bulls (Table S1). A total of 10 Mb was sequenced per targeted region, 5 Mb upstream and 5 Mb downstream of the most significant SNP marker (Fig. 1). Library preparation and subsequent targeted DNA sequencing were performed by RAPiD Genomics. Figure S1 describes in detail the different steps of the variant calling workflow. Briefly, quality control of the raw sequencing reads was performed using fastqc (v0.11.7; Babraham Bioinformatics). Reads were mapped to the bovine reference genome ARS‐UCD1.2 using burrows wheeler aligner (v0.7.17; BWA‐MEM algorithm; Li & Durbin 2009). PCR duplicates were identified and removed using picard tools (v2.19.1, Broad Institute). Local realignment around insertions/deletions (InDels) was carried out using the Genome Analysis Toolkit (gatk, v3.8.1; McKenna *et al*. 2010). First, *RealignerTargetCreator* was used to identify what regions needed to be realigned, and then *IndelRealigner* was used to perform the actual realignment. Base quality score recalibration was applied to recalibrate read quality scores. Single nucleotide variants (SNVs) and InDels were identified using *HaplotypeCaller* algorithm in GATK. The program vcftools (v0.1.16; Danecek *et al*. 2011) was used to call SNPs and InDels and extract the variants in each targeted segment of the genome. Finally, SNVs and InDels were classified based on their location on the genome using Variant Effect Predictor tool (version 92; McLaren *et al*. 2010). The consequences of amino acid substitutions on protein function were predicted using the SIFT tool (Ng & Henikoff 2003). Gene expression profiles of genes harboring strong candidate causal variants were obtained from NCBI, using *Homo sapiens*, *Mus musculus*, and *Ovis aries*.

Table S2 provides a summary of the sequencing read alignments. The sequencing depth per pool varied from 39× to 51× in high‐fertility pools and from 40× to 48× in low‐fertility pools. The mapping coverage was 99.99% for both high‐ and low‐fertility pools. About 120k SNVs and more than 18k InDels were detected across the five targeted regions. As expected, most of these genetic variants were identified in intergenic and intronic regions, while a small proportion was annotated in genic regions, including exons and untranslated regions. A total of 7144 of the 120k SNVs showed opposing homozygous genotypes between low‐ and high‐fertility pools (File S1). Interestingly, among these 7144 SNVs, 74 were missense mutations and three were nonsense mutations (Table 1). By contrast, of the 18k InDels identified, 852 showed opposing genotypes between low‐ and high‐fertility pools (File S2). Of special interest, out of 852 InDels, five were classified as frameshift variants, while two were classified as in‐frame variants.

**Table 1:** List of candidate causal single nucleotide variants.

Chr	Location	SNP name	High SCR	Low SCR	Gene	Codon	AA	SIFT
8	71516867	rs441998010	GG	AA	*ADAM28*	Ggg/Agg	Gly/Arg	0.02
9	40620329	rs457222030	AA	GG	*AK9*	Att/Gtt	Ile/Val	0.12
13	59672426	rs448912315	CC	TT	*SIRPD*	Cgt/Tgt	Arg/Cys	0.18
13	61455628	rs453494838	CC	GG	*TTLL9*	Ctc/Gtc	Leu/Val	0.01
13	61867061	rs381304038	GG	AA	*ASXL1*	Gcc/Acc	Ala/Thr	0.11
17	63363385	rs453223261	GG	AA	*TCHP*	aCg/aTg	Thr/Met	0.01
17	63835900	rs109993813	CC	TT	*FOXN4*	tCg/tTg	Ser/Leu	0.01
27	34092466	rs109302554	AA	GG	*PLEKHA2*	gAt/gGt	Asp/Gly	0.01

*BTA8 (67.2–77.2 Mb):* Bulls in the low‐fertility pool carried two missense mutations, located on genes *ENTPD4* (rs110428633, SIFT = 0.10) and *ADAM28* (rs441998010, SIFT = 0.02). Gene *ADAM28* is part of the ADAM family, and although its function is not well‐characterized, members of the ADAM family are membrane‐anchored proteins implicated in a variety of biological processes involving cell–cell and cell–matrix interactions, including sperm–egg interactions (Edwards *et al*. 2008). In addition, low‐fertility bulls also carried an in‐frame deletion mutation (rs434538966) located in gene *NEFM*. This gene encodes a neurofilament protein and is expressed exclusively in brain, adrenal gland, and testis (Lariviere & Julien 2004).

*BTA9 (38.7–48.7 Mb):* Bulls in the low‐fertility pool carried a missense mutation (rs457222030, SIFT = 0.12) located in gene *AK9*. Notably, *AK9* is highly expressed in testis (Fig. 2), and is involved in maintaining the homeostasis of cellular nucleotides by catalyzing the interconversion of nucleoside phosphates (Amiri *et al*. 2013). Low‐fertility bulls also carried a frameshift mutation (rs380835932) located in gene *AMD1*, which encodes an important intermediate enzyme in polyamine biosynthesis and is ubiquitously expressed in many tissues (Pegg 2009).

**Figure 2:**
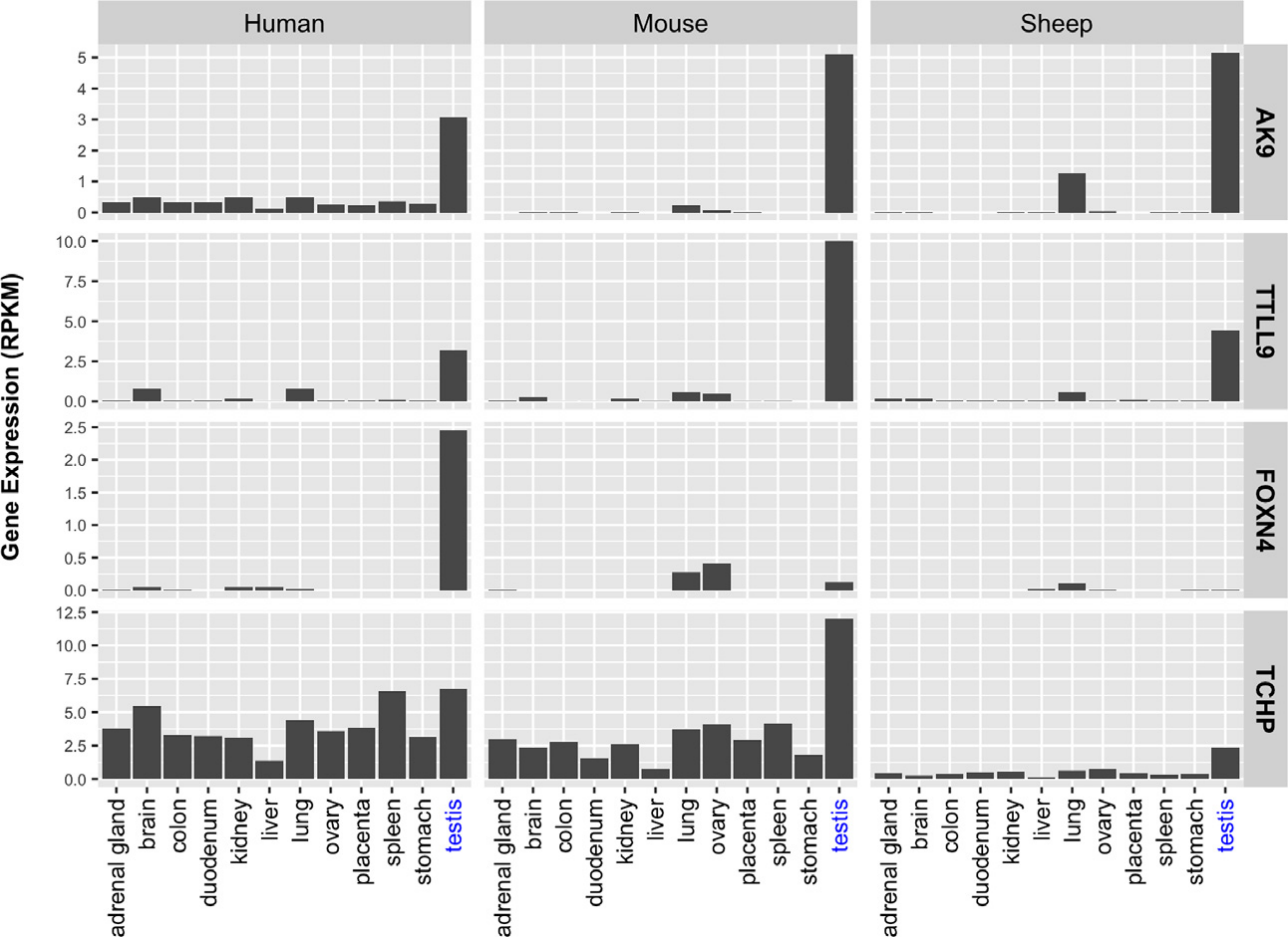
Gene expression profiles of genes that carry strong candidate causal variants. Gene expression profiles were obtained from NCBI.

*BTA13 (55.2–65.2 Mb):* Two nonsense mutations (stop gained) and 13 missense mutations were identified in this genomic region. The two nonsense mutations were located in two novel cattle genes, *ENSBTAG00000052136* and *ENSBTAG00000048009*. Moreover, some of the most deleterious missense mutations carried by low‐fertility bulls were located in genes exclusively or highly expressed in testis, such as *TTLL9* (rs453494838, SIFT = 0.01), *SIRPD* (rs448912315, SIFT = 0.18), and *ASXL1* (rs381304038, SIFT = 0.11; Fig. 2). Gene *TTLL9* is a testis‐specific gene that plays essential roles in sperm motility (Konno *et al*. 2016). Bulls in the low‐fertility pool also carried an in‐frame insertion (rs523959552) in gene *SOX12*, a member of the SOX family of transcription factors that are expressed in various tissues (Sarkar & Hochedlinger 2013).

*BTA17 (58.3–68.3 Mb):* A total of 25 missense mutations were identified, the vast majority considered as tolerant amino acid substitutions (high SIFT values). The two most remarkable SNVs were rs453223261 and rs109993813, two very intolerant mutations (SIFT = 0.01) carried by low‐fertility bulls on genes *TCHP* and *FOXN4*, respectively. Gene *TCHP* is highly expressed in testis (Fig. 2), it encodes a keratin‐binding protein that is involved in cytoskeleton organization, including negative regulation of cilium assembly (Martello *et al*. 2020). Gene *FOXN4* encodes a member of the winged‐helix/forkhead family of transcription factors, a group of proteins that display a remarkable functional diversity and are involved in a wide variety of biological processes (Carlsson & Mahlapuu 2002). Notably, in humans, *FOXN4* is exclusively expressed in testis (Fig. 2). In addition, low‐fertility bulls carried a stop gained mutation in gene *OAS2* and frameshift variants in genes *TCHP* and *OAS1X*. Note that neither *OAS2* nor *OAS1X* are directly implicated in male fertility, nor exclusively/highly expressed in testis; both genes encode members of the 2‐5A synthetase family, proteins involved in the innate immune response (Mozzi *et al*. 2015).

*BTA27 (29.7–39.7 Mb):* Five missense mutations were identified in this region. Low‐fertility bulls carried one deleterious mutation (rs109302554, SIFT = 0.01) in *PLEKHA2*, a ubiquitously expressed gene implicated in phosphatidylinositol biosynthetic process (Dowler *et al*. 2000). The other four missense mutations, all considered as tolerant amino acid substitutions, were located in members of the ADAM family, namely *ADAM2*, *ADAM3A*, and *ADAM9*. Interestingly, genes *ADAM2* and *ADAM3A* are exclusively expressed in testis, and play important roles in sperm‐egg interactions (Cho 2012).

Note that the presence of copy number variants (CNVs) within the targeted regions was also assessed, resulting in the detection of 36 CNVs (data not shown). Since they were mostly located in intergenic regions, and considering the limitations of pooling and targeted sequencing in the accurate evaluation of structural variants, we focused our analysis on candidate SNVs and InDels.

In summary, we have identified a set of high‐impact mutations in low‐fertility bulls, including nonsense, missense, and frameshift variants. Some of these mutations may be considered as strong candidate causal variants for bull subfertility, given its deleterious effects and its location in genes directly implicated in male reproduction (Table 1). Our findings suggest that, for at least some of the targeted regions, it is likely that not one, but several causal variants are responsible for the observed phenotypic effects. Future studies should validate our results. This work is the foundation for the development of novel genomic tools that will allow early detection and culling of subfertile dairy bull calves.

## Supporting information

**Figure S1** Variant calling workflow.

**Table S1** Descriptive statistics of Holstein bulls used in the targeted sequencing.

**Table S2** Summary statistics of targeted sequencing data.

**File S1** SNVs with opposing homozygous genotypes between low‐ and high‐fertility pools.

**File S2** InDels with opposing genotypes between low‐ and high‐fertility pools.

## Data Availability

DNA‐Sequencing data can be accessed through NCBI SRA PRJNA714249.
