# Supplementary material for: Targeted sequencing reveals candidate causal variants for dairy bull subfertility
Source: Anim Genet. 2021 May 24;52(4):509–13. doi: 10.1111/age.13089 (PMC8361668; doi:10.1111/age.13089)
Supplement: Supplementary file 1 — Figure S1 Variant calling workflow. [file AGE-52-509-s004.pdf]

VARIANT CALLING WORKFLOW

1 PROCESSING RAW READS

Check the quality of the raw sequence data

Tool: FastQC
Input: fastq
Output: Report

2 MAPPING READS

Map the reads against the reference genome

Tool: BWA
Input: fastq
Output: SAM

3 PROCESSING READ ALIGNMENTS

Convert SAM to BAM; then sort
Mark duplicates
Add read groups
Reorder

Tool: SAMtools, PIcard
Input: SAM
Output: BAM

4 QUALITY SCORE RECALIBRATION

Create Realignment Targets
Realign Indels
Quality Score Recalibration

Tool: GATK
Input: BAM
Output: BAM

5 SNP CALLING

Call Variants
Joint Variant Calling
Extract SNPs & Indels
Filter SNPs & Indels

Tool: GATK
Input: BAM
Output: VCF

6 VARIANT ANNOTATION

Predict variant function

Tool: VEP
Input: VCF
Output: txt
