# Supplementary material for: Targeted sequencing reveals candidate causal variants for dairy bull subfertility
Source: Anim Genet. 2021 May 24;52(4):509–13. doi: 10.1111/age.13089 (PMC8361668; doi:10.1111/age.13089)
Supplement: Supplementary file 2 — Table S1 Descriptive statistics of Holstein bulls used in the targeted sequencing. [file AGE-52-509-s001.docx]

| **Group** | **Target region**  **(Mb)** | **N** | **Mean SCR**  **(min, max)** | **Mean breedings**  **(min, max)** |
| --- | --- | --- | --- | --- |
| Low SCR | BTA08: 67.2-77.2 | 22 | -7.1 (-10.1, -5.2) | 1320 (649, 3536) |
|  | BTA09: 38.7-48.7 | 08 | -9.2 (-17.1, -3.0) | 834 (363, 1254) |
|  | BTA13: 55.3-65.3 | 23 | -7.5 (-15.8, -4.9) | 1208 (347, 3004) |
|  | BTA17: 58.3-68.3 | 20 | -6.5 (-13.6, -4.4) | 1150 (466, 2886) |
|  | BTA27: 29.7-39.7 | 14 | -7.1 (-11.0, -4.2) | 1345 (376, 4243) |
| High SCR | Five regions | 22 | +4.5 (+2.7, +5.9) | 6309 (881, 87180) |
| SCR: Sire Conception Rate | | | | |

**Table S1.** Descriptive statistics of Holstein bulls used in the targeted sequencing.
