# Supplementary material for: Targeted sequencing reveals candidate causal variants for dairy bull subfertility
Source: Anim Genet. 2021 May 24;52(4):509–13. doi: 10.1111/age.13089 (PMC8361668; doi:10.1111/age.13089)
Supplement: Supplementary file 3 — Table S2 Summary statistics of targeted sequencing data. [file AGE-52-509-s002.docx]

| **Group** | **Target region (Mb)** | **Number**  **unique reads** | **Depth** | **Coverage (%)** |
| --- | --- | --- | --- | --- |
| Low SCR | BTA08: 67.2-77.2 | 4,635,852 | 48.36X | 99.99 |
|  | BTA09: 38.7-48.7 | 3,341,920 | 40.40X | 99.99 |
|  | BTA13: 55.3-65.3 | 5,326,660 | 47.17X | 99.99 |
|  | BTA17: 58.3-68.3 | 3,750,094 | 44.31X | 99.99 |
|  | BTA27: 29.7-39.7 | 3,425,763 | 47.68X | 99.99 |
| High SCR | BTA08: 67.2-77.2 | 4,339,568 | 51.46X | 99.99 |
|  | BTA09: 38.7-48.7 | 3,911,810 | 46.48X | 99.99 |
|  | BTA13: 55.3-65.3 | 3,992,162 | 47.17X | 99.99 |
|  | BTA17: 58.3-68.3 | 3,300,191 | 39.33X | 99.99 |
|  | BTA27: 29.7-39.7 | 4,325,262 | 51.15X | 99.99 |

**Table S2.** Summary statistics of targeted sequencing data.
